# Global use of asbestos - legitimate and illegitimate issues

**DOI:** 10.1186/s12995-020-00267-y

**Published:** 2020-06-15

**Authors:** Arthur L. Frank

**Affiliations:** Dornsife School of Public Health of Drexel University, 3215 Market Street, 7th Floor, Philadelphia, PA 19104 USA

**Keywords:** Asbestos, Chrysotile, Amphibole, “Safe use”, Fiber toxicology

## Abstract

**Background:**

Exposure to asbestos causes non-malignant and malignant diseases including asbestosis, lung cancer, and mesothelioma. The modern history of such diseases goes back more than a century.

**Main text:**

While much is known about the ability of asbestos to cause disease, the carcinogenic mechanism is not yet understood. Continuing legitimate scientific questions include such issues as potential differential toxicity and carcinogenicity of different fiber types. Illegitimate issues include the supposed “safe” use of asbestos, and the chrysotile hypothesis.

**Conclusion:**

Asbestos disease issues are highly politicized and vested economic interests perpetuate false issues regarding the hazards of asbestos.

## Background

Asbestos is a term that applies to two families of naturally occurring fibrous minerals, the first family being the amphiboles (amosite, crocidolite, anthophyllite, actinolite, tremolite) and the single member of the serpentine minerals family (chrysotile) both having fibers that have a structure of length to width of at least 3 to 1 and fibers can range from very small (1 μm in length) to those too large to be inhaled into the lungs. Each of the six fiber types is chemically distinct. At the present time, because of past use and experience, only the chrysotile form of asbestos, also called white asbestos, is currently being used, with some very few minor exceptions. Over time, chrysotile has made up some 90–95% of all the asbestos that has ever been used around the world. The only commercially important amphiboles were amosite and crocidolite.

Although there is a long history of asbestos use going back thousands of years, the modern era of utilization of asbestos starts about 125 years ago. Previously widely used in many products, there are some 3000–4000 or more prior uses of asbestos in many products, most no longer being made [1]. Common uses for asbestos have included construction materials, brakes of many types, asbestos textiles, and large quantities were used aboard ocean-going vessels. A convention regarding ships essentially stopped the use of asbestos in new vessels as of 2002. At its peak, some 800 thousand tons of asbestos per year was utilized in the United States in the early 1970s, but usage in the developed world has markedly decreased. Now less than one thousand tons of raw fiber are brought into the United States on an annual basis. Some uncommon prior uses of asbestos include being found as a filtering agent for drugs and wine, its mixture with plastics for long-playing records, and use in consumer products such as hair dryers and bowling balls. Currently, more than 60 nations have completely banned the use of asbestos and this includes virtually all industrialized nations except for the United States. Even Canada, previously a major world supplier, has banned the use of asbestos, but must now fight against a desire of some to make money from processing asbestos waste to extract minerals. Current production is still around 2 million tons, most coming from Russia, and about half is utilized in two countries alone – China and India. Other developing countries continue to use considerable amounts of asbestos [2]. In addition to asbestos from Russia, other countries such as Kazakhstan and China also mine considerable amounts. Brazil, a country with a long history of mining, became the first country with active mines to ban asbestos, followed recently by Colombia. The United Nations has attempted to label chrysotile asbestos an extremely hazardous product in international commerce, but this has been blocked by a number of countries for many years, including Russia, India, and others. Furuya and colleagues have reported on the prevalence of asbestos disease worldwide [3], with some yearly 250,000 deaths estimated.

## Main text

Some 2000 years ago the Romans were aware of the potential health hazards of asbestos and recognized that slaves mining this material were at a serious health risk. The modern era of asbestos being appreciated as being hazardous to health started in the late 1800s with an official report from the government of the United Kingdom [4]. Over time the world has learned that there are two major classes of disease following exposure to asbestos, the non-malignant diseases, as well as the many cancers caused by asbestos.

The non-malignant diseases include asbestos warts, an insignificant problem, arising when asbestos gets into the skin. The usual first manifestation after exposure to asbestos, also uncommon, is the finding of a benign asbestoic pleural effusion. This is a condition characterized by bloody fluid in the chest, raising concern about a possible malignancy, but none is found. This often occurs within the first 10 years of exposure. The most common manifestations of asbestos-related disease, other than the cancers, is the finding of asbestosis, one of the pneumoconioses. This is a condition characterized by scarring in the lung and also of the pleura, the tissue lining around the lung and pericardium.

Traditionally, going back to the work of Zenker [5] who coined the term “pneumoconiosis”, or dust disease of the lung in 1876, fibrotic changes of both the parenchyma and pleura were characteristic of this class of disease. Biologically, it is the same cells putting down the same type of scar tissue containing collagen that can occur both in the lungs or in the pleura that characterize these fibrotic changes which are permanent when they occur. Although Murray wrote in 1907 [6] of a young man developing fibrosis after working with asbestos textiles, the term asbestosis was first coined by Cooke in 1924 [7] and the work of Lanza [8] discussed both the parenchymal and pleural aspects of asbestosis. Selikoff [9] continued to recognize the term asbestosis as meaning either parenchymal asbestosis, pleural asbestosis, or a combination of both. It is only relatively recently that some have split off pleural asbestosis and are now more likely to call it asbestos-related pleural disease. What one calls this disease is irrelevant, but it should be recognized that the scarring phenomena is biologically and historically the same. Although a benign condition, asbestosis can lead to death and among insulators, the most heavily exposed occupational group that is generally known, approximately 10% of insulators have over time died of asbestosis [10].

Without question, it is agreed that there are some cancers that are definitively linked to prior exposures to asbestos. This includes lung cancer, mesothelioma, a cancer of tissue linings, ovarian cancer, and laryngeal cancer. Other cancers appear to have increasingly supportive data as being linked to exposure to asbestos and this would include a range of gastrointestinal tract cancers, including esophagus [11], stomach [12], and colorectal cancers [13]. Also, other oropharyngeal cancers [14] have been linked to exposures to asbestos, as have kidney cancers [15]. Taken together, between the non-malignant causes of death from asbestos as well as the malignancies, in a group such as insulators, about 50% of all workers will ultimately die of some asbestos-related condition.

All asbestos related diseases have a dose-response relationship, and it is the cumulative exposure, from all fiber types, that must be said to cause disease. Clearly, with multiple sources of exposure all exposures contribute, although some exposures may contribute more and some less. All exposures increase the risk of developing disease, although clearly not everyone exposed will develop disease.

When considering asbestos-related disease one must keep in mind both the concept of time since first onset of exposure, as well as the concept of length of exposure. The first time, often called latency, refers to when is one first exposed to a carcinogen such as asbestos, and when do such diseases arise. There is a concept known as the “twenty-year rule” whereby most, although not all, cancers caused by occupational exposures will first arise in significant numbers after about 20 years from first exposure. With regard to asbestos, cancers begin to appear as soon as 10 years, with some rare cases even earlier, but they begin to peak after 20 years reaching a high point in the 35–45-year range. They can continue to occur even later in life. The second concept, time of exposure, gets to the concept of dose. The question becomes “how much asbestos does it take to cause cancer”. There is evidence in the scientific literature in both animals [16] and humans [17] that as little as 1 day of exposure can give rise to mesotheliomas, as well as lung cancers arising after short exposures. The work of Selikoff [18] documents a doubling of the risk of lung cancer with as little as a month of exposure to asbestos. This finding of quite short exposures to asbestos gives legitimacy to the concept that there appears to be no threshold that should be recognized for carcinogens, applying this to asbestos just as the American Petroleum Institute applied this to the exposures to benzene as expressed in the late 1940s when it was stated that the only safe level of exposure to benzene would be zero. In 2014 the Occupational Safety and Health Administration in the US again noted on their website that there was no evidence of a threshold below which disease could not appear. In the 1960s two asbestos company physicians felt there was no level of exposure to asbestos that could not produce harm.

Much of the understanding of the diseases caused by asbestos have been well proven by epidemiologic studies. However, it should be noted that epidemiological investigations are not needed to link exposure to a substance and the subsequent development of disease. We have learned this from a number of relatively modern diseases such as vinyl chloride causing angiosarcomas of the liver, and the world outbreaks of Severe Acute Respiratory Syndrome (SARS) and Middle East Respiratory Syndrome (MERS). That said, for asbestos the recognition that it can cause lung cancer preceded epidemiological studies, and the first definitive statement that asbestos could cause lung cancer attributed to Hueper [19] did not require proof or justification with the use of epidemiology. This came later with the work of other such as Doll [20]. Similarly, the work of Wagner in 1960 also showed, without an epidemiologic study, the relationship of exposure to asbestos and the development of mesothelioma [21].

For those groups that have been studied epidemiologically, there have been none in which evidence of disease has not been found. This includes not only many occupational groups such as shipyard workers, insulators, and other construction workers, brake mechanics, among others, but also family members of workers can develop disease from para-occupational exposures, and even true environmental exposures have led to malignant and non-malignant disease. It should be recognized that it is absolutely not necessary to do an epidemiological study among each group, nor each product, to document that asbestos can cause illness in any group. This concept was well recognized by Selikoff many decades ago [22]. He made it clear that it was exposure to asbestos, not the characterization of the person, or setting of exposure, that was paramount.

The problem of mesothelioma, which had been suggested with individual case studies in the scientific literature prior to the publication of Wagner [21], was now fully recognized after Wagner presented his data of the dozens of cases seen by him in just a few short years, all emanating from the asbestos mines of the North West Cape Province in South Africa.

As noted above, many countries have totally banned the use of asbestos, and some have done so far enough in the past, for some 30 years or more, that evidence exists that the cessation of the use of asbestos has been characterized by a decrease in the number of new mesothelioma cases. A problem with asbestos is that there is an extremely long latency, especially after short-term exposures, and only after some 50 or more years of a total worldwide ban would one anticipate there to be no further health issues related to asbestos, except for the disturbance of the millions of tons still in place, if not removed correctly.

As is true for many scientific matters, there are legitimate continuing scientific questions regarding asbestos. What has characterized asbestos disease, along with a few other exposures, such as the data generated over tobacco smoking and lead exposure, is that in some quarters what has been called “doubt science” has generated information regarding illegitimate scientific questions [23]. There are some issues that should fully be recognized as being settled, although some legitimate scientific issues may still exist.

Among the legitimate scientific questions regarding exposure to asbestos would be the matter of the relative toxicity of the different fiber types. There is a continuing controversy over the potential level of toxicity between amphiboles and chrysotile, and some data show that a mixture of the two has a synergistic affect. The other matter of some legitimate concern is the question of fiber size going back to the work of Stanton [24]. Stanton showed that fibers of a particular size appear to be more carcinogenic than others but the groups of animals he studied were small, the method of asbestos application not one resembling true human exposure, and the difficulty of doing such research because of the problem of getting accurately sized aliquots of asbestos fibers remains one that has been little studied since this early work. Of note, however, is that he did not consider, as some do, only fibers above five microns as being biologically active or carcinogenic.

Among the controversies regarding asbestos that should be completely set aside is, first, that chrysotile does not cause some of the asbestos-related diseases. Among the claims that have been made over the years is that chrysotile cannot cause mesothelioma, that chrysotile cannot cause peritoneal mesothelioma, that chrysotile from certain products cannot cause disease, or that only extraordinarily large exposures to chrysotile can cause disease. There has been a tawdry history of industry paying scientists for some of these opinions. Another controversy with little real support is that only fibers above five microns can cause disease. While it is true that in the United States traditionally only those fibers five microns or larger were counted, the reason is not because of supposed biological inactivity of smaller fibers, but that this was the optical threshold for light microscopy, and when standards were put in place not enough electron microscopes were available to accurately measure smaller fibers. Many scientists [25–27] have documented the predominance of short chrysotile fibers in cases of mesothelioma, contradicting the thought that chrysotile is not associated with the development of mesothelioma. There is even support in the literature that “pure” chrysotile can cause mesothelioma [28, 29].

Another fallacy, arising primarily from pathologists, is that one must actually see asbestos fibers in tissue, or some number of asbestos bodies. This view neglects recognizing that chrysotile fibers much less commonly than amphiboles create asbestos bodies, and most asbestos that has been used has been chrysotile. Requiring a certain amount of fibers in tissue, for which there is no standardized test method allowing gross manipulation of the findings, also does not recognize the much more rapid disappearance of chrysotile in tissue compared to the amphiboles.

This also puts into question the whole notion of “biopersistance” as having any significant role in judging what can cause disease. As noted above, far more chrysotile is found in the pleura, thus making claims of its absence in the lung as a sign of not producing disease faulty. Finding more amphibole in tissue does not mean chrysotile could not have produced disease.

Another fallacy is the belief that underlying asbestosis must be present to relate a cancer to asbestos exposures. Different cell types are involved and in other settings, one does not need silicosis to say lung cancer is caused by silica.

An additional fallacy is that there is such a thing as the “safe use” of chrysotile asbestos. This is a sham put forward by industry and never has been documented, even in the most advanced developed countries. To claim this flies in the face of worldwide data and experience. No country has ever documented safe use. One way used to claim “safe use” is to ignore the recording of mesotheliomas, as has been done in Russia and elsewhere. As is well appreciated in public health, the absence of data does not mean the absence of disease. There were many pleural and peritoneal mesotheliomas documented in the former East Germany where large amounts of Russian chrysotile were used in the past.

Another controversy, put forth by the Indian asbestos industry, has been that the genetic makeup of the Indian population is such that Indians do not develop asbestos-related disease, this occurring only in westerners. To support such statements, it is cited that no case of mesothelioma has ever been recorded in India. To counteract this lack of official government recognition of disease, it should be noted that at one hospital in India alone, in 1 year, there were 32 cases of mesothelioma being treated with obviously none ever recorded officially at that time by the government [2]. While earlier ICD codes for disease did not separately list mesothelioma, such separate listing is now available through the ICD-10 coding system, but in some countries, mesotheliomas are not being separately recorded, as noted above. Issues of experience with histopathologic testing and lack of experience by most pathologists helps explain the underreporting of mesotheliomas, but does not explain everything.

When one considers the economic issues related to asbestos, both in countries where use continues or in those countries where litigation has been prominent, one can understand why false scientific information would be forthcoming. In countries such as India, prominent individuals tend to own asbestos cement facilities, and have seen that tariffs on asbestos are kept low while tariffs on safer substitutes make such substitutes economically unviable. In places like the United States, many billions of dollars have been and will be at stake with regard to asbestos-related litigation, and some insurance entities are especially known for making payments difficult, or holding on to adjudicated payments so as to increase their income off the “float” of funds that they have on hand. As has been stated about the field of occupational medicine, there is probably no more political field within medicine, and within that specialty the world of asbestos is among the most contentious. Millions of dollars have been spent, sometimes on very specific products, such as brakes, to create doubt [23].

Clearly, many dozens of countries, now more than sixty, have realized that they can function perfectly well without the use of asbestos, not putting their citizenry at risk. Many developing countries continue to use asbestos, claiming that it is cheap and useful, and downplay the hazard to miners, workers who use it, and to the general population. Somewhat harder to understand is the continued legal use of asbestos in countries such as the United States where interference with government regulatory activities has taken place making it hard, if not impossible, to ban the use of this unneeded material as has happened in so many other places. With political leadership not recognizing the hazards of asbestos exposure, even calling the matter a “Chinese hoax”, little leadership can be expected from some quarters.

In some countries it is noted that the United States has not banned the use of asbestos, and the rhetorical question then becomes, “why should we”.

Some countries have had partial bans in place, but commercial entities have found ways of getting around such regulatory activities and although use may have been limited to certain settings, the easy availability of material may have a more widespread utilization in reality [30].

It is unfortunate that in those very countries where significant amounts of asbestos continue to be used, the healthcare systems are totally inadequate for taking care of large numbers of people with significant diseases such as lung cancer, mesothelioma, and others that are caused by asbestos. Ironically, the synergy between asbestos and cigarette smoking causing excess numbers of lung cancers, the number being far greater than the simple additive effect of those two carcinogenic exposures, [31] is taking place in countries where some commercial entities are pushing for the continued use of asbestos, while other commercial entities are pushing for the increasing use of tobacco products.

## Conclusions

Just as the public health community has worked to rid the globe of preventable disease, as we have seen with smallpox, and almost with polio, there is no reason why the world could not, going forward, become asbestos free. There would still be the lingering hazards from the millions of tons of asbestos currently in place, including in waste piles, and the many products in commerce that currently contain asbestos. However, if one could at some point totally ban the new use of asbestos on a global scale some 50 years thereafter there would be much less asbestos disease being seen, and going forward, with no more continued use and careful removal and disposal thereafter one could eliminate asbestos-related disease and its 250,000 yearly deaths from the world.

Complete total bans would not be an easy matter because of continuing vested economic interests, and the continuing development of “doubt science” and the economic benefit to certain entities and individuals from work testifying on behalf of asbestos manufacturers. That said, it can only be hoped that over time the truth about asbestos and disease, and the benefits of cessation, will be recognized and acted upon. One must overcome sometimes similar economic and political forces, but hopefully scientific truths and recognition of the significant hazards posed by asbestos will bring about complete cessation of use.

## Data Availability

Not applicable.

## Abbreviations

SARS: Severe Acute Respiratory Syndrome
MERS: Middle East Respiratory Syndrome
